# Cost-effectiveness of Population-Wide Genomic Screening for Hereditary Breast and Ovarian Cancer in the United States

**DOI:** 10.1001/jamanetworkopen.2020.22874

**Published:** 2020-10-29

**Authors:** Gregory F. Guzauskas, Shawn Garbett, Zilu Zhou, Scott J. Spencer, Hadley S. Smith, Jing Hao, Dina Hassen, Susan R. Snyder, John A. Graves, Josh F. Peterson, Marc S. Williams, David L. Veenstra

**Affiliations:** 1 The Comparative Health Outcomes, Policy & Economics (CHOICE) Institute, Department of Pharmacy, University of Washington, Seattle; 2 Department of Biostatistics, Vanderbilt University, Nashville, Tennessee; 3 Department of Health Policy, Vanderbilt University Medical Center, Nashville, Tennessee; 4 Institute for Public Health Genetics, University of Washington, Seattle; 5 Center for Medical Ethics and Health Policy, Baylor College of Medicine, Houston, Texas; 6 Department of Population Health Sciences, Geisinger, Danville, Pennsylvania; 7 Department of Health Policy and Behavioral Sciences, Georgia State University, Atlanta; 8 Department of Biomedical Informatics, Vanderbilt University Medical Center, Nashville, Tennessee; 9 Genomic Medicine Institute, Geisinger, Danville, Pennsylvania

## Abstract

**Importance:**

Genomic screening for hereditary breast and ovarian cancer (HBOC) in unselected women offers an opportunity to prevent cancer morbidity and mortality, but the potential clinical impact and cost-effectiveness of such screening have not been well studied.

**Objective:**

To estimate the lifetime incremental incidence of HBOC and the quality-adjusted life-years (QALYs), costs, and cost-effectiveness of HBOC genomic screening in an unselected population vs family history–based testing.

**Design, Setting, and Participants:**

In this study conducted from October 27, 2017, to May 3, 2020, a decision analytic Markov model was developed that included health states for precancer, for risk-reducing mastectomy (RRM) and risk-reducing salpingo-oophorectomy (RRSO), for earlier- and later-stage HBOC, after cancer, and for death. A complimentary cascade testing module was also developed to estimate outcomes in first-degree relatives. Age-specific RRM and RRSO uptake probabilities were estimated from the Geisinger MyCode Community Health Initiative and published sources. Parameters including RRM and RRSO effectiveness, variant-specific cancer risk, costs, and utilities were derived from published sources. Sensitivity and scenario analyses were conducted to evaluate model assumptions and uncertainty.

**Main Outcomes and Measures:**

Lifetime cancer incidence, QALYs, life-years, and direct medical costs for genomic screening in an unselected population vs family history–based testing only were calculated. The incremental cost-effectiveness ratio (ICER) was calculated as the difference in cost between strategies divided by the difference in QALYs between strategies. Earlier-stage and later-stage cancer cases prevented and total cancer cases prevented were also calculated.

**Results:**

The model found that population screening of 30-year-old women was associated with 75 (95% credible range [CR], 60-90) fewer overall cancer cases and 288 QALYs (95% CR, 212-373 QALYs) gained per 100 000 women screened, at an incremental cost of $25 million (95% CR, $21 millon to $30 million) vs family history–based testing; the ICER was $87 700 (78% probability of being cost-effective at a threshold of $100 000 per QALY). In contrast, population screening of 45-year-old women was associated with 24 (95% CR, 18-29) fewer cancer cases and 97 QALYs (95% CR, 66-130 QALYs) gained per 100 000 women screened, at an incremental cost of $26 million (95% CR, $22 million to $30 million); the ICER was $268 200 (0% probability of being cost-effective at a threshold of $100 000 per QALY). A scenario analysis without cascade testing increased the ICER to $92 600 for 30-year-old women and $354 500 for 45-year-old women. A scenario analysis assuming a 5% absolute decrease in mammography screening in women without a variant was associated with the potential for net harm (−90 QALYs per 100 000 women screened; 95% CR, −180 to 10 QALYs).

**Conclusions and Relevance:**

The results of this study suggest that population HBOC screening may be cost-effective among younger women but not among older women. Cascade testing of first-degree relatives added a modest improvement in clinical and economic value. The potential for harm conferred by inappropriate reduction in mammography among noncarriers should be quantified.

## Key Points

**Question:**

Is it cost-effective to implement population-wide genomic screening for hereditary breast and ovarian cancer (HBOC)?

**Findings:**

This decision analytical model study found that genomic screening for HBOC among unselected women may be cost-effective depending on the age distribution of the women screened. Cascade testing of first-degree relatives added a modest improvement in clinical and economic value.

**Meaning:**

Population-level genomic screening for HBOC targeting women aged 20 to 35 years could be considered in settings in which the outcomes of screening can be evaluated, particularly to avoid a reduction in mammography screening among patients with negative test results.

## Teaser

This decision analysis estimates the lifetime incremental incidence of hereditary breast and ovarian cancer and the quality-adjusted life-years, costs, and cost-effectiveness of hereditary breast and ovarian cancer genomic screening in an unselected population vs family history–based testing.

## Introduction

Hereditary breast and ovarian cancer (HBOC) genetic variants, particularly in the *BRCA1* (OMIM 113705) and *BRCA2* (OMIM 600185) genes, confer increased lifetime risk of developing cancer in women.^1^ Knowledge of HBOC variants empowers women to choose more intensive precancer screening practices, including magnetic resonance imaging (MRI) to detect cancer sooner, and for some to undergo chemoprevention and/or prophylactic risk-reducing mastectomy (RRM) and/or risk-reducing salpingo-oophorectomy (RRSO), which lower cancer risk and mortality.^2,3^

However, many HBOC carriers are identified only after their first cancer diagnosis, often owing to a lack of knowledge or an unremarkable family history that did not warrant genomic testing according to current guidelines.^4,5^ A landmark study of population-based screening in the Ashkenazi Jewish population of Israel, a known population at high risk of HBOC, showed that 50% of families with HBOC sequence variations would be missed using family history–based testing alone^6^; this finding was later confirmed in a US population study.^7^ Consequently, it has been argued that population-based screening of unselected women for HBOC is needed to address the gap in cancer prevention left by the current policy of personal history–based testing or family history–based testing.^8^

The Centers for Disease Control and Prevention lists testing for HBOC syndrome as a tier 1 genomic application, with “significant potential for positive impact on public health based on available evidence-based guidelines and recommendations,”^9^ but, to date, the US Preventive Services Task Force guidelines for *BRCA1/2* testing recommend against routine risk assessment, genetic counseling, or genetic testing for women whose personal or family history does not suggest HBOC carrier status.^10^

Population-wide HBOC screening thus presents several important policy questions. Of 42 million US women aged 30 to 49 years,^11^ an estimated 250 000 to 415 000 (0.6%-1%) have actionable *BRCA1/2* variants.^6^ Would the health benefits associated with population-based genomic screening conferred to a relatively small number of HBOC variant carriers justify the cost required of such a significant public health endeavor? At under what circumstances should women typically be screened given the complexities of health care coverage and reproductive choices? And at what age could the potential benefits associated with genomic screening become negligible or even harmful?

Initial cost-effectiveness analyses have suggested that HBOC population screening is cost-effective for women aged 25 to 30 years.^12,13^ However, these studies did not explicitly model age-based cancer risk, gradual intervention uptake, or clinical outcomes over time. Previous analyses also have not considered the impacts of cascade testing, wherein relatives of HBOC variant carriers identified by screening can be informed and offered the opportunity to undergo testing. Last, the potential harms of screening to noncarriers—primarily the encouragement of a false sense of well-being—have not been previously explored, to our knowledge. Our objective was to estimate the health outcomes and economic cost of a US population screening program for HBOC variants in an unselected population using an age-based decision model, a novel cascade testing modeling approach, and extensive scenario analyses.

## Methods

### Primary Population Screening Model

**Figure 1.**
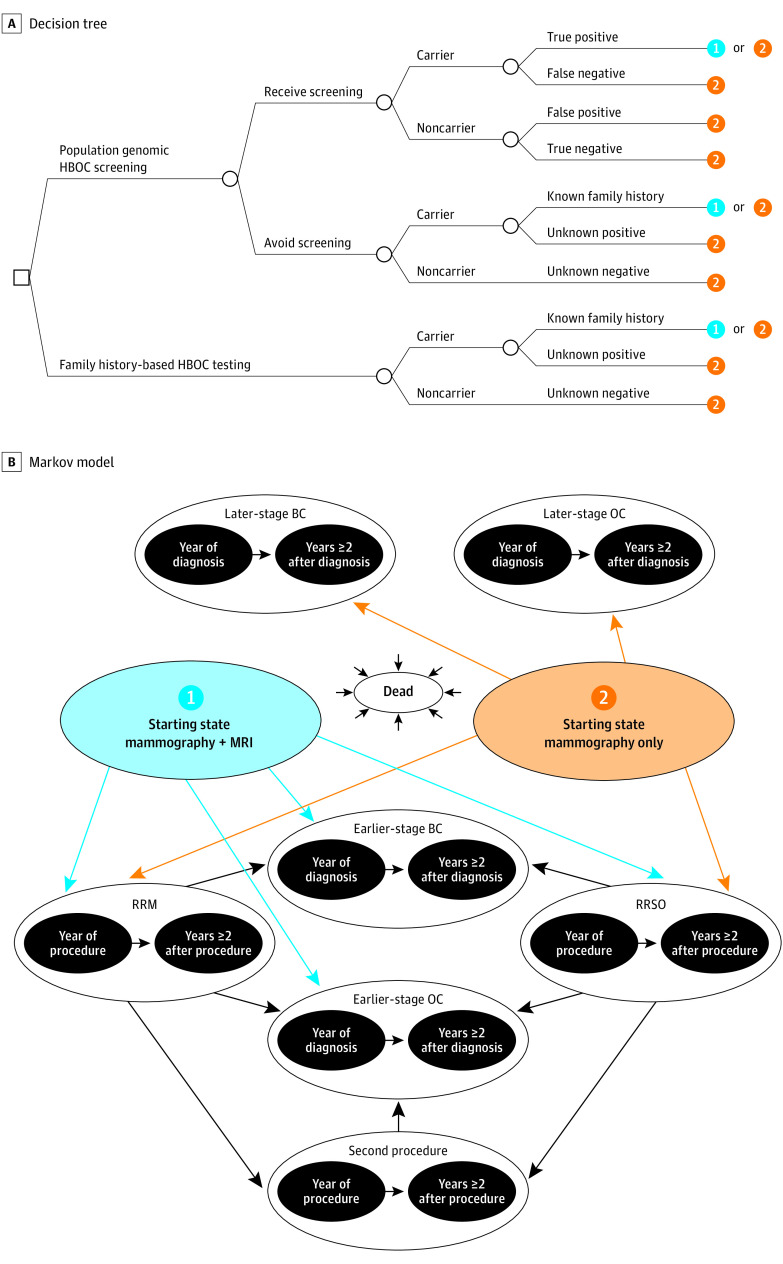
Decision Model A, Decision tree. B, Markov model. 1 Indicates mammography plus magnetic resonance imaging (MRI); 2, mammography only; BC, breast cancer; HBOC, hereditary breast and ovarian cancer; OC, ovarian cancer; RRM, risk-reducing mastectomy; and RRSO, risk-reducing salpingo-oophorectomy.

In this decision analytical model study conducted from October 27, 2017, to May 3, 2020, we developed a decision tree plus Markov model to compare population screening for HBOC in an unselected population of previously undiagnosed women vs no population screening; routine family history–based testing (ie, the status quo policy) was available in both strategies (Figure 1). The decision tree was used to distribute individuals by carrier or noncarrier and known or unknown variant status among Markov health states in the first model cycle. The Markov model was used to model individuals’ health care actions, clinical events, and health care costs over a lifetime. We used a US health care sector perspective (ie, focused on direct medical care costs only) and discounted all cost and health outcomes by 3% per year.^14^ The model was developed in Microsoft Excel (Microsoft Corp). Approval from the University of Washington institutional review board was not required because the study used secondary data sources.

The following clinical events were modeled in the decision tree: (1) screening participation on the part of the individual, (2) carrier or noncarrier status, (3) screening results (parameterized by test sensitivity and specificity) and/or family history–based testing results, and (4) specific pathogenic gene variants, including *BRCA1, BRCA2, ATM* (OMIM 607585), *CHEK2* (OMIM 604373), *MSH6* (OMIM 600678), *PALB2* (OMIM 610355), *RAD51C* (OMIM 602774), and *TP53* (OMIM 191170) (gene variants not shown in Figure 1). The long-term Markov model included health states for precancer monitoring (mammography plus MRI for known carriers who choose it [starting state 1] or mammography only [starting state 2]), 1-year health states (single-cycle health states used to assess specific temporary transitions, costs, and/or utilities) for incidence of early-stage or late-stage breast or ovarian cancer, after early-stage or late-stage breast or ovarian cancer, 1-year health states for RRM and RRSO procedures, after RMM and after RRSO, after RRM plus RRSO, and all-cause death. We used annual model cycles, and patients could remain in their current state or transition to another state each year as depicted by the arrows in Figure 1. All patients could transition to death from any other health state based on cancer-specific and/or background mortality.^15,16,17^

### Cascade Testing Module

We developed an accompanying module for cascade testing of family members of HBOC variant carriers identified by population-based screening (eFigure 1 in the Supplement). The cascade testing tree calculations were dynamically tied to the primary population screening model in that the number of surviving mothers, sisters, and daughters was dependent on the modeled age of patients entering the primary model. Cascade testing module outcomes included testing cost among tested individuals and the incremental cost and quality-adjusted life-year (QALY) outcomes generated by newly identified carriers through the cascade testing process. These outcomes were then added back into the primary population screening model to calculate combined overall results (eFigure 2 in the Supplement).

### Clinical Parameters

**Figure 2.**
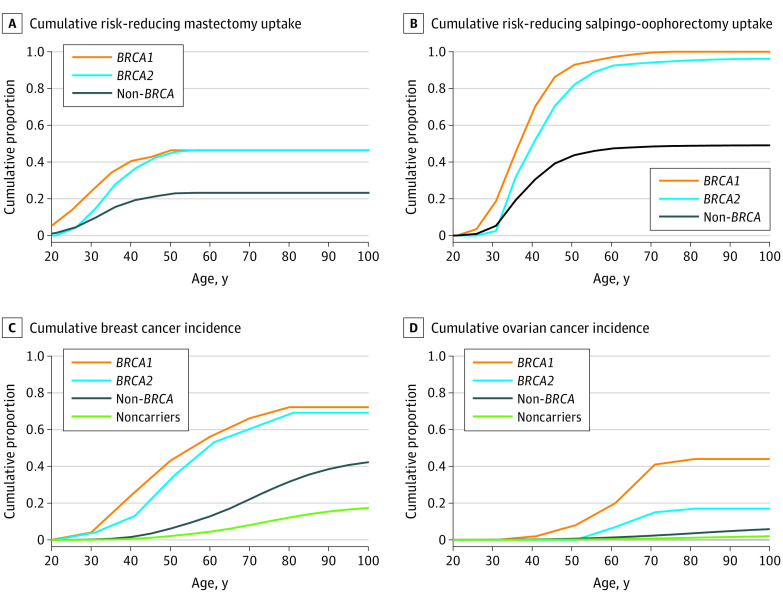
Modeled Lifetime Cumulative Surgery Uptake and Cancer Incidence A, Cumulative risk-reducing mastectomy uptake.^31^ B, Cumulative risk-reducing salpingo-oophorectomy uptake.^31^ C, Cumulative breast cancer incidence.^1,16,17^ D, Cumulative ovarian cancer incidence.^1,16,17^

The overall prevalence of pathogenic HBOC variant carriers (0.5%) was based on data from the Geisinger MyCode Community Health Initiative, a health care system–based genomic medicine research project with more than 260 000 patient participants in Pennsylvania and New Jersey (Table 1).^7,18^ Annualized, age-based RRM and RRSO uptake probabilities were primarily derived from published cumulative estimates by Chai et al,^31^ who observed a prospective cohort of 1499 women with inherited *BRCA1/2* mutations but without prior cancer or RRM or RRSO from 20 clinical and research genetics centers in the Prevention and Observation of Surgical Endpoints (PROSE) consortium^32^ (Figure 2^1,16,17,31^). The PROSE consortium findings were supported by a small sample of RRM and RRSO uptake data from the Geisinger MyCode Community Health Initiative, which found that the RRM uptake during the first year of follow-up was 3.5% (2 of 57) and that the RRSO uptake was 11.8% (6 of 51) among known pathogenic *BRCA1/2* carriers.^33^ We did not identify comparable prophylactic surgery uptake data for non-*BRCA1/2* variants, so we assumed RRM and RRSO uptake rates of 50% of the averaged uptakes of *BRCA1* and *BRCA2* carriers.

**Table 1.** Model Parameters

Parameter	Base case (lower-upper)	Distribution	Source
Mutation prevalence			
Hereditary breast and ovarian cancer carrier prevalence (overall), % (±20%)	0.495 (0.452-0.538)	Beta	Manickam et al^7^ and Dewey et al^18^
Proportion: *BRCA1*	0.276 (0.221-0.332)	Dirichlet	Kurian et al^19^
Proportion: *BRCA2*	0.290 (0.232-0.348)	Dirichlet	Kurian et al^19^
Proportion: *ATM*	0.121 (0.096-0.145)	Dirichlet	Kurian et al^19^
Proportion: *CHEK2*	0.145 (0.116-0.174)	Dirichlet	Kurian et al^19^
Proportion: *MSH6*	0.042 (0.034-0.051)	Dirichlet	Kurian et al^19^
Proportion: *PALB2*	0.091 (0.073-0.109)	Dirichlet	Kurian et al^19^
Proportion: *RAD51C*	0.027 (0.022-0.032)	Dirichlet	Kurian et al^19^
Proportion: *TP53*	0.008 (0.006-0.009)	Dirichlet	Kurian et al^19^
Decision tree, % (±20%)			
Carriers identified through family history–based testing	0.174 (0.139-0.209)	Beta	Manchanda et al^13^
Screening test			
Sensitivity (±20%)	0.991 (0.793-1.000)	Beta	Manchanda et al^13^
Specificity (±20%)	0.999 (0.799-1.000)	Beta	Toland et al^20^
Proportion who avoid screening (±20%)	0.050 (0.040-0.060)	Beta	Assumption
Proportion of known carriers who undergo magnetic resonance imaging (±20%)	0.750 (0.600-0.900)	Beta	Assumption
Non-*BRCA* mutation breast cancer risk, OR (95% CI)			
* ATM*	2.97 (1.67-5.68)	Log normal	Lu et al^21^
* CHEK2*	2.19 (1.40-3.56)	Log normal	Lu et al^21^
* MSH6*	2.59 (1.35-5.44)	Log normal	Lu et al^21^
* PALB2*	5.53 (2.24-17.65)	Log normal	Lu et al^21^
Non-*BRCA* mutation ovarian cancer risk, OR (95% CI)			
* ATM*	2.85 (1.30-6.32)	Log normal	Lu et al^21^
* MSH6*	4.16 (1.95-9.47)	Log normal	Lu et al^21^
* RAD51C*	18.38 (14.70-184.00)	Log normal	Lu et al^21^
* TP53*	18.50 (2.56-808.10)	Log normal	Lu et al^21^
Risk-reducing interventions			
Breast cancer after mastectomy, HR (95% CI)			
* BRCA1*	0 (No residual risk)	NA	Domchek et al^2^
* BRCA2*	0 (No residual risk)	NA	Domchek et al^2^
Non-*BRCA*	0 (No residual risk)	NA	Assumption
Breast cancer after oophorectomy, HR (95% CI)			
* BRCA1*	0.63 (0.41-0.96)	Log normal	Domchek et al^2^
* BRCA2*	0.36 (0.16-0.82)	Log normal	Domchek et al^2^
Non-*BRCA*	0.36 (0.16-0.82)	Log normal	Assumption
Ovarian cancer after oophorectomy, HR (95% CI)			
* BRCA1*	0.31 (0.12-0.82)	Log normal	Domchek et al^2^
* BRCA2*	0 (No residual risk)	NA	Domchek et al^2^
Non-*BRCA*	0 (No residual risk)	NA	Assumption
Mortality, % (±20%)			
Breast cancer mortality risk reduction: early stage	0.943 (0.900-1.000)	Log normal	Evans et al^22^
Breast cancer 5-y relative mortality			
Aged <45 y	0.119 (0.095-0.143)	Log normal	SEER program^16^
Aged 45-54 y	0.094 (0.075-0.113)	Log normal	SEER program^16^
Aged 55-64 y	0.099 (0.079-0.119)	Log normal	SEER program^16^
Aged 65-74 y	0.086 (0.069-0.103)	Log normal	SEER program^16^
Aged ≥75 y	0.129 (0.103-0.155)	Log normal	SEER program^16^
Ovarian cancer 5-y relative mortality			
Aged <45 y	0.235 (0.188-0.282)	LogNormal	SEER program^16^
Aged 45-54 y	0.409 (0.327-0.491)	Log normal	SEER program^16^
Aged 55-64 y	0.505 (0.404-0.606)	Log normal	SEER program^16^
Aged 65-74 y	0.613 (0.490-0.736)	Log normal	SEER program^16^
Aged ≥75 y	0.791 (0.633-0.949)	Log normal	SEER program^16^
Background mortality	US life tables	NA	CDC^15^
Intervention uptake: non-*BRCA* mutations (±20%)			
Rate ratio vs combined *BRCA*: mastectomy	0.500 (0.400-0.600)	Log normal	Assumption
Rate ratio vs combined *BRCA*: oophorectomy	0.500 (0.400-0.600)	Log normal	Assumption
Quality of life, % (±20%)			
Utility: breast cancer	0.663 (0.530-0.796)	Beta	Peasgood et al^23^
Utility: ovarian cancer	0.628 (0.502-0.754)	Beta	Manchanda et al^13^
Utility: after breast cancer	0.810 (0.648-0.972)	Beta	Manchanda et al^13^
Utility: after ovarian cancer	0.720 (0.576-0.864)	Beta	Havrilesky et al^24^
Disutility (1 y): mastectomy	0.030 (0.024-0.036)	Beta	Li et al^25^
Disutility (1 y): oophorectomy	0.030 (0.024-0.036)	Beta	Li et al^25^
Disutility (1 y): knowledge of variant	0.050 (0.040-0.060)	Beta	Li et al^25^
Disutility: screening harm to noncarriers (scenario analysis)	0.030 (0.029-0.032)	Beta	Mandelblatt et al^26^
Risk-reducing intervention costs, mean (±20%), $			
Mastectomy	22 110 (17 688-26 532)	Normal	Sun et al^27^
Oophorectomy	8476 (6781-10 171)	Normal	Sun et al^27^
Mammography	228 (182-274)	Normal	Sun et al^27^
Intense screening (magnetic resonance imaging)	1403 (1122-1683)	Normal	Sun et al^27^
Screening assay	200 (160-240)	Normal	Color Genomics^28^
Confirmation	200 (160-240)	Normal	Color Genomics^28^
Cancer treatment costs, mean (±20%), $			
Aged <65 y			
Breast cancer—initial	83 633 (66 906-100 360)	Normal	Sun et al^27^
Breast cancer—continuing	8048 (6438-9658)	Normal	Sun et al^27^
Breast cancer—last year	68 022 (54 418-81 626)	Normal	Sun et al^27^
Ovarian cancer—initial	133 121 (106 497-159 745)	Normal	Sun et al^27^
Ovarian cancer—continuing	14 635 (11 708-17 562)	Normal	Sun et al^27^
Ovarian cancer—last year	93 005 (74 404-111 606)	Normal	Sun et al^27^
Aged ≥65 y			
Breast cancer—initial	83 633 (66 906-100 360)	Normal	Sun et al^27^
Breast cancer—continuing	8048 (6438-9658)	Normal	Sun et al^27^
Breast cancer—last year	68 022 (54 418-81 626)	Normal	Sun et al^27^
Ovarian cancer—initial	133 121 (106 497-159 745)	Normal	Sun et al^27^
Ovarian cancer—continuing	14 635 (11 708-17 562)	Normal	Sun et al^27^
Ovarian cancer—last year	93 005 (74 404-111 606)	Normal	Sun et al^27^
Cascade testing parameters, % (±20%)			
Proportion identified carriers who inform family	0.70 (0.56-0.84)	Beta	Roberts et al^29^ and Elrick et al^30^
Proportion of family members ever tested	0.20 (0.16-0.24)	Beta	Roberts et al^29^ and Elrick et al^30^

Abbreviations: CDC, Centers for Disease Control and Prevention; HR, hazard ratio; NA, not applicable; OR, odds ratio; SEER, Surveillance, Epidemiology, and End Results.

We used the *BRCA1/2*-specific cumulative cancer risk from Kuchenbaecker et al,^1^ who conducted a prospective cohort study of 6036 *BRCA1* and 3820 *BRCA2* female carriers from the International *BRCA1/2* Carrier Cohort Study, the Breast Cancer Family Registry, and the Kathleen Cuningham Foundation Consortium for Research into Familial Breast Cancer (Figure 2^1,16,17,31^). The cumulative risks were used to derive annual breast and ovarian cancer transition probabilities for *BRCA1* and *BRCA2* carriers; *BRCA1* and *BRCA2* carriers were modeled independently.

Based on another PROSE consortium^32^ publication, we modeled the RRM- and RRSO-associated cancer risk reduction using hazard ratios from Domchek et al,^2^ who conducted a prospective study of 2482 women with *BRCA1* or *BRCA2* mutations at 22 clinical and research genetics centers. We assumed that the outcomes among non-*BRCA* variant carriers who underwent RRM or RRSO had cancer risk reductions equal to that of *BRCA2* carriers.

### Quality-of-Life (Utility) Parameters

We assumed a health state utility of 1.0 for healthy patients receiving mammography with or without MRI. The utilities for breast and ovarian cancer (year 1 and years ≥2) were obtained from the published literature.^13,23,24^ We assigned disutilities for RRM and RRSO during the year of the surgery.^25^ Previous models have explored a 1-year disutility of 0.08 to 0.13 associated with learning one’s carrier status of a pathogenic variant^34,35^; we assumed a 1-year disutility of 0.05 in the first model cycle to recognize the potential psychological impact of receiving a positive screening result.

### Cost Parameters

All costs were in 2019 US dollars. We modeled a population screening test cost of $200 based on the testing options currently available to the public.^28^ The costs for mammography, MRI, and prophylactic procedures were derived from published sources.^13,27^ Cancer costs were assessed for the first year of treatment, for continuing treatment in subsequent years, and as palliative therapy for the last year of life.^27^ We assumed that the transition to death from any 1-year or after cancer health state incurred palliative treatment costs.

### Statistical Analysis

We calculated lifetime cancer incidence, QALYs, life-years, and direct medical costs for genomic screening in an unselected population vs family history–based testing only. The incremental cost-effectiveness ratio (ICER) was calculated as the difference in cost between strategies divided by the difference in QALYs between strategies. We also calculated earlier-stage and later-stage cancer cases prevented and total cancer cases prevented. We report results for 30-year-old and 45-year-old women only; a broader range of outcomes for women of different modeled ages is available in the eTable in the Supplement.

We performed a scenario analysis that did not include the effects of cascade testing. Separately, we evaluated the potential harm to noncarriers (ie, the potential for noncarriers to decrease adherence to mammography screening after receiving a negative genomic screening result); this evaluation was modeled as a discounted lifetime disutility of −0.03% applied to 5% of screened noncarriers.^26^ We also performed 1-way and probabilistic sensitivity analyses to assess the association of uncertainty in model parameters with the results. In 1-way sensitivity analysis, 1 parameter at a time is varied to its low and high value while keeping all other parameters constant; in probabilistic sensitivity analysis, all model parameters were simultaneously randomly varied according to an assigned probability distribution over 5000 simulations, and 95% credible ranges (CRs) were calculated for each model result.

## Results

### Base Case

**Figure 3.**
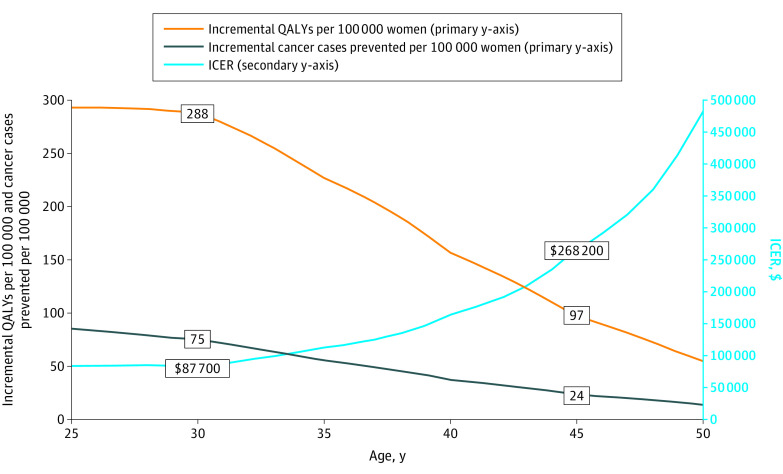
Base Case Results by Age of Genomic Screening ICER indicates incremental cost-effectiveness ratio; and QALYs, quality-adjusted life-years.

Screening 30-year-old women was associated with 75 (95% CR, 60-90) fewer overall cancer cases per 100 000 women, with 138 additional cases of early-stage cancer but 214 fewer cases of late-stage cancer per 100 000 women (Table 2). Screening for HBOC was associated with 288 QALYs (95% CR, 212-373 QALYs) gained per 100 000 women screened, at an incremental cost of $25 million (95% CR, $21 million to $30 million) vs family history–based testing, which was associated with an ICER of $87 700 (Figure 3). In contrast, screening 45-year-old women was associated with 24 (95% CR, 18-29) fewer cancer cases per 100 000 women, with 159 additional cases of early-stage cancer but 183 fewer cases of late-stage cancer. We calculated 97 QALYs (95% CR, 66-130 QALYs) gained, at an incremental cost of $26 million (95% CR, $22 million to $30 million), which was associated with an ICER of $268 200.

**Table 2.** Model Results for Ages 30 and 45 Years

Characteristic	Cases/100 000			Cost/woman screened, $	Quality-adjusted life years/woman screened	Life-years/woman screened	ICER, $
	Early-stage cancer	Late-stage cancer	Total cancer				
**Screened at age 30 y**							
Population screening, base case without cascade testing (95% CR)	143 (111 to 172)	13 642 (12 897 to 14 363)	13 786 (13 040 to 14 512)	14 400 (13 100 to 15 900)	25.7990 (25.6830 to 25.9219)	25.9200 (25.8297 to 26.0395)	NA
Family history–based testing, base case without cascade testing (95% CR)	26 (19 to 34)	13 827 (13 083 to 14 555)	13 853 (13 109 to 14 583)	14 200 (12 800 to 15 600)	25.7964 (25.6799 to 25.9198)	25.9179 (25.8268 to 26.0382)	NA
Incremental							
Base case without cascade testing (95% CR)	117 (90 to 141)	−184 (−210 to −152)	−67 (−82 to −51)	240 (200 to 290)	0.0026 (0.0015 to 0.0038)	0.0022 (0.0011 to 0.0032)	No cascade testing: 92 600
Cascade testing (95% CR)	21 (14 to 30)	−29 (−41 to −20)	−8 (−12 to −5)	9 (6 to 13)	0.0003 (0.0002 to 0.0004)	0.0002 (0.0001 to 0.0003)	With cascade testing: 87 700
**Screened at age 45 y**							
Population screening, base case without cascade testing (95% CR)	169 (124 to 203)	12 762 (12 024 to 13 499)	12 930 (12 198 to 13 672)	18 500 (16 800 to 20 300)	21.7245 (21.5895 to 21.8776)	21.8706 (21.7607 to 22.0169)	NA
Family history–based testing, base case without cascade testing (95% CR)	31 (22 to 40)	12 916 (12 181 to 13 657)	12 947 (12 213 to 13 686)	18 300 (16 600 to 20 100)	21.7238 (21.5883 to 21.8768)	21.8698 (21.7597 to 22.0167)	NA
Incremental							
Base case without cascade testing (95% CR)	138 (100 to 167)	−154 (−184 to −114)	−16 (−20 to −10)	250 (210 to 290)	0.0007 (0.0000 to 0.0014)	0.0008 (0.0000 to 0.0016)	No cascade testing: 354 500
Cascade testing (95% CR)	21 (14 to 30)	−29 (−41 to −19)	−8 (−11 to −5)	10 (7 to 14)	0.0003 (0.0002 to 0.0004)	0.0002 (0.0001 to 0.0003)	With cascade testing: 268 200

Abbreviations: CR, credible range; ICER, incremental cost-effectiveness ratio; NA, not applicable.

### Scenario Analyses

#### Cascade Testing

At the population level, the incremental associations of cascade testing were modest; when we excluded cascade testing, the incremental cost and QALYs gained per screened 30-year-old woman decreased by approximately $900 000 and 25, respectively. For a 45-year-old woman, the incremental cost and QALYs gained decreased by approximately $1 000 000 and 27, respectively. Removing cascade testing from the population screening model was associated with an increased overall ICER of $92 600 for 30-year-old women and $354 500 for 45-year-old women (Table 2).

#### Potential Harm

When we considered the potential harm of genomic screening to noncarriers, who might avoid recommended mammography after being informed of a negative result, the net harm was −90 QALYs (95% CR, −180 to 10 QALYs). Consequently, the incremental QALYs of population screening decreased from 288 to 146 (95% CR, 65-250) per screened 30-year-old woman, which increased the ICER to $172 700. For 45-year-old women, the incremental QALYs decreased from 97 to −44 (95% CR, −85 to −1), which was associated with population screening being dominated by family history–based testing.

### Sensitivity Analyses

In the 1-way sensitivity analysis for 30-year-old women, the ICER was most sensitive to 5-year breast cancer survival, screening assay cost, cancer risk reductions after RRSO, and RRM and RRSO uptake (eFigure 3 in the Supplement). For 45-year-old women, the ICER was most sensitive to 5-year breast cancer survival, RRM and RRSO uptake, the mortality reduction applied to early-stage breast cancer, screening assay cost, and HBOC carrier prevalence.

Probabilistic sensitivity analysis results are presented as cost-effectiveness acceptability curves, which show the bayesian probability that the results are cost-effective at increasing willingness to pay per QALY thresholds (eFigure 4 in the Supplement). For 30-year-old women, population screening had a 0%, 78%, and 100% probability of being cost-effective vs family history–based testing at the $50 000, $100 000, and $150 000 per QALY thresholds, respectively. When we added potential harm to noncarriers who avoid recommended mammography, the probability of population screening being cost-effective decreased to 0%, 2%, and 34% at the $50 000, $100 000, and $150 000 per QALY thresholds, respectively. Population screening was not cost-effective for 45-year-old women in any probabilistic simulations with a willingness to pay per QALY below $150 000.

## Discussion

We conducted a cost-effectiveness analysis to estimate whether the lifetime reductions in HBOC incidence and the QALYs gained by implementing a population genomic screening program in an unselected population warrant its additional cost compared with family history–based testing only. Our model was informed by age-specific uptake rates of RRM and RRSO and their effectiveness at preventing HBOC, age-specific HBOC incidence, variant-specific HBOC risk, and the associations of cascade testing. We found that population genomic screening is moderately cost-effective for younger women, that cascade testing adds some but not fundamentally impactful clinical and economic value, and that the potential harm conferred by screening noncarriers should be considered.

Our analysis accounted for the dynamics of age and health choice timing by incorporating the findings of the Geisinger MyCode Community Health Initiative and the PROSE-based study by Chai et al,^31^ who found that, while fewer than half of *BRCA1/2* mutation carriers underwent RRM in their lifetimes, most RRM procedures occurred before 50 years of age. Most carriers eventually underwent RRSO; however, many did so after the recommended period (prior to 40 years of age or after completion of childbearing).^31^ These findings underscore our conclusion that screening younger populations is more cost-effective because most RRM and RRSO procedures occur prior to 50 years of age, while most cases of HBOC occur after 50 years of age. Older newly identified carriers thus have a smaller window in which to act, which limits the potential benefits associated with screening.

The associatons of cascade testing for HBOC have not been previously estimated, to our knowledge. At the population level, we found that the incremental associations of cascade testing were modest. This finding was associated primarily with the small number of identified carriers relative to noncarriers, the proportion of carriers who tell their family members that they are carriers (70%), and the proportion of informed family members who receive testing (20%).^29,30^ Nonetheless, cascade testing should be implemented where feasible, and efforts should be made to improve the rates of family communication and follow-up testing because a higher uptake of cascade testing should improve the overall value of population genomic screening.

Our scenario analysis assuming a decrease in mammography screening in women without an HBOC variant was associated with the potential for net harm (−90 QALYs per 100 000 women screened), making population genomic screening not cost-effective for 30-year-old women. Thus, population-level screening for HBOC should be conducted in settings in which the outcomes of screening can be evaluated, particularly mammography screening for women who have negative test results or who do not receive a positive result if negative results are not returned. Furthermore, additional studies on the potential harms of population screening and on how to keep informed noncarriers engaged in recommended health behaviors are warranted.

Our results differed notably from those of previous studies. Manchanda et al^13^ estimated that population genomic screening of 30-year-old women gained 0.007 incremental QALYs (2.7 quality-adjusted days) per woman screened compared with family history–based testing, whereas we estimated 0.003 incremental QALYs (1.05 quality-adjusted days) per woman screened, a 2.6-fold difference. A previous study reported that this difference is owing to the prior study’s use of a decision tree–only model structure, which likely overestimates the association of HBOC screening by not accounting for competing risks over time.^36^ For example, by assessing the lifetime cumulative uptake of RRM and RRSO all at once instead of gradually over time, a decision tree may overestimate the reduction in lifetime cancer incidence by abruptly shrinking the available pool of women at risk. Another study, by Zhang et al,^12^ found that HBOC screening would be associated with health care cost savings and prevention of disability-adjusted life-years (DALYs) and would be highly cost-effective, with an ICER of $12 973 per DALY prevented. By recreating the decision model in the study, we were able to reproduce the number of breast cancer cases prevented but not the benefits (73.3 DALYs per case prevented and 121.8 DALYs per death prevented).^37^ In their response, Lacaze et al^38^ countered that these high values reflect the benefits associated with preventing and delaying the onset of nonfatal cancers (breast and colorectal cancers); however, this response does not explain how the per-person benefits exceed human life expectancy, which is difficult to interpret.

### Limitations

Our analysis has some limitations. First, we identified a wide range of supporting literature for cascade testing parameters regarding informing family members and subsequent testing rates; thus, the most appropriate estimates were difficult to discern. However, an informal review of Geisinger MyCode Community Health Initiative uptake rates among family members of newly identified carriers supported our current estimates. Second, we had limited data on adherence to general population breast cancer screening guidelines among HBOC variant carriers. We assumed that 75% of known carriers choose to undergo regular MRI screening in addition to mammography, and when we varied this assumption in 1-way sensitivity analysis (eFigure 3 in the Supplement), MRI uptake was not nearly as impactful as uptake of RRM and RRSO. Thus, the uptake of risk-reducing surgical procedures is the key factor associated with absolute benefit, particularly RRSO given the comparatively high mortality of ovarian vs breast cancer, rather than baseline adherence to mammography.

Third, our analysis did not directly evaluate the impacts of patient diversity and health care disparities. The 2019 US Preventive Services Task Force guidelines on *BRCA* screening highlighted the need for more data on variant prevalence and the downstream impacts on different ethnicities.^10^ African American women, for example, have been shown to have similar to higher incidence of *BRCA1/2* mutations compared with White women but are comparatively more likely to be diagnosed at a younger age and with later-stage cancer, are less likely to be offered genetic testing, and have lower rates of risk-reducing surgery.^39,40^ Focused analyses of racial/ethnic subgroups and other underserved populations, such as African American individuals, represent an important follow-up opportunity.

Fourth, false positives were not considered other than our assumption that a confirmation test (with associated cost) would correct the screening error; recent media coverage of prophylactic surgery cases for false-positive *BRCA1/2* test results highlight that significant harm may occur to a small number of screened individuals. Fifth, we lacked data on RRM and RRSO uptake for non-*BRCA1/2* variants and thus assumed that uptake was 50% for *BRCA1/2*; 1-way sensitivity analysis showed that the associations of the assumption were minimal given the lower prevalence of these mutations. Sixth, our model considered population genomic screening for HBOC only, whereas a national screening program would likely include other tier 1 genomic syndromes,^9^ which would likely improve the clinical and economic value. Ultimately, the true value of HBOC screening should be assessed within the context of a broader screening panel capable of identifying multiple diseases within the general population.

## Conclusions

We found that population genomic screening is moderately cost-effective for younger women, with a special emphasis on targeting women aged 20 to 35 years. In addition, we found that cascade testing is important for achieving a true population-level reach but is not fundamentally associated with the clinical and economic value and that the potential harm conferred by screening noncarriers should be considered.

## Notes

Supplement. **eAppendix.** Additional Methods and Expanded Results **eFigure 1.** Cascade Testing Module Decision Tree **eFigure 2.** Calculation of Cascade Testing Outcomes **eFigure 3.** Results of One-Way Sensitivity Analysis **eFigure 4.** Results of Probabilistic Sensitivity Analysis **eTable.** Expanded Model Results **eReferences.**
