# Supplementary material for: Cost-effectiveness of Population-Wide Genomic Screening for Hereditary Breast and Ovarian Cancer in the United States
Source: JAMA Netw Open. 2020 Oct 29;3(10):e2022874. doi: 10.1001/jamanetworkopen.2020.22874 (PMC7596578; doi:10.1001/jamanetworkopen.2020.22874)
Supplement: Supplement. — eAppendix. Additional Methods and Expanded Results eFigure 1. Cascade Testing Module Decision Tree eFigure 2. Calculation of Cascade Testing Outcomes eFigure 3. Results of One-Way Sensitivity Analysis eFigure 4. Results of Probabilistic Sensitivity Analysis eTable. Expanded Model Results eReferences. [file jamanetwopen-e2022874-s001.pdf]

Supplementary Online Content

Guzauskas GF, Garbett S, Zhou Z, et al. Cost-effectiveness of population-wide genomic screening for hereditary breast and ovarian cancer in the United States. *JAMA Netw Open.* 2020;3(10):e2022874. doi:10.1001/jamanetworkopen.2020.22874

eAppendix. Additional Model Methods and Expanded Results
eFigure 1. Cascade Testing Module Decision Tree
eFigure 2. Calculation of Cascade Testing Outcomes
eFigure 3. Results of One-Way Sensitivity Analysis
eFigure 4. Results of Probabilistic Sensitivity Analysis
eTable. Expanded Model Results
eReferences.

This supplementary material has been provided by the authors to give readers additional information about their work.

eAppendix. Additional Model Methods and Expanded Results

Decision Tree Parameters

The overall prevalence of pathogenic HBOC variant carriers (0.5%) was based on data from the Geisinger MyCode Community Health Initiative, a Geisinger system-wide DNA biobank with more than 190,000 patient-participants in Pennsylvania and New Jersey.[1, 2] We used test sensitivity (99%) and specificity (99%) to calculate the likelihood that tested individuals are correctly identified.[3] False positives were not considered other than our assumption that a confirmation test (with associated cost) would correct the screening error. The proportion of HBOC variants identified with family history testing was 17.4%, based on a prior cost-effectiveness analysis of HBOC population screening versus family history testing alone.[4] We then used a recent database analysis of 95,561 women who were tested for HBOC using a next generation sequencing panel to calculate the proportions of HBOC variants that were *BRCA1, BRCA2, ATM, CHEK2, MSH6, PALB2, RAD51C,* and *TP53*.[5]

Markov Model Transitions

A proportion of pre-cancer, known carriers who opted to undergo intensive screening entered the Markov model in starting state 1, in which they received mammography plus MRI according to age-based guidelines for high risk individuals.[6, 7] Pre-cancer (1) noncarriers, (2) known carriers who opted out of intensive screening, and (3) unknown

© 2020 Guzauskas GF et al. *JAMA Network Open.*

carriers entered the Markov model in starting state 2, in which they received mammography only according to age-based guidelines for average risk individuals.[8, 9] Known carriers could opt for prophylactic RRM or RRSO based on age-based cumulative uptake among BRCA1/2 positive women (Figure 2 in main text).[10] We modeled a 1-year health state for the year of the procedure, wherein we applied procedure costs and disutilities (Table 1 in main text). Women who underwent RRM or RRSO then transitioned to post-RRM or post-RRSO procedure health states with reduced risks of cancer incidence.[11] Individuals with one procedure could transition to another 1-year state for a second procedure using a weighted probability of RRM and RRSO, with weighted procedure costs and disutilities; these patients then transitioned to a post-2nd procedure health state where we assumed their breast cancer risk was zero, but the age-based, RRSO-adjusted probability of ovarian cancer remained.[11] We assumed the small number of individuals who receive RRM and/or RRSO and nonetheless go on to develop cancer transition to earlier stage cancers due to continued intensive screening.

All individuals were at risk for breast or ovarian cancer according to age-based cancer incidence among carriers and non-carriers (Figure 2 in main text).[12-14] We assumed women undergoing intensive mammography plus MRI would be diagnosed at an earlier stage, on average, than those undergoing standard mammography based on a study showing there were significantly fewer patients with positive lymph nodes at the time of cancer removal surgery in the MRI-screened group compared to the mammography-only screened group.[15] We assumed equivalent incidence of earlier versus later stage

© 2020 Guzauskas GF et al. *JAMA Network Open.*

breast or ovarian cancer, however we applied a mortality risk reduction to earlier stage breast cancer health states over the individual's remaining lifetime.[15] In both the earlier and later stage cancer states, we modeled first year-specific treatment costs and utility values. Patients who survived the first year of breast or ovarian cancer then transitioned to post-breast or post-ovarian cancer health states with long-term/continuing treatment costs and utility values.

We also derived age-based estimates of non-*BRCA* variant (*ATM*, *CHEK2*, *MSH6*, *PALB2*, *RAD51C*, *TP53*) cancer incidence from Lu et al., who conducted whole-exome sequencing and gene-phenotype associations on a sample of 11,416 patients with clinical features of breast cancer, ovarian cancer, or both from 1200 hospitals and clinics across the United States, plus 3988 controls who were referred for genetic testing for noncancer conditions.[13] Odds ratios from Lu et al. of breast and/or ovarian cancer risk were converted to relative risk estimates and applied to the cancer incidence data derived for the noncarrier population. Non-*BRCA* mutations were modeled in the Markov model as an HBOC variant prevalence-weighted pooled group. Annual age-based cancer incidence among noncarriers was derived from the Surveillance, Epidemiology, and End Results (SEER) Program.[14]

Breast Screening Uptake

We based uptake of mammography and MRI on current guidelines.[6-9] Noncarriers and unknown carriers were assumed to undergo routine mammography according to

© 2020 Guzauskas GF et al. *JAMA Network Open.*

guidelines for average risk women; starting at age 40, we modeled that 50% chose to receive optional annual mammography, increasing to the recommended 100% from ages 45-54, then all women received recommended biannual mammography from age 55 until death. Known carriers were assumed to undergo routine mammography according to guidelines for increased risk women, which recommend annual mammography alternated with annual breast MRI every 6 months. We assumed 75% of women with known increased risk opted for intensive screening with MRI in addition to mammography. We further assumed the remainder of known increased risk women received mammography only at the recommended frequency.

Cascade Testing Module

We used a decision tree to organize key elements of cascade testing including (a) the probability a newly identified carrier will inform their family members, (b) the number of living first degree female relatives, (c) the probability that informed relatives will opt to undergo testing, (d) carrier/noncarrier status, and (e) testing and/or family history testing result. The probabilities for (a) and (c) were informed by a review of family communication of genetic results studies;[16, 17] based on our findings, we assumed that 70% of population screening identified carriers go on to inform their family members, and 20% of family members go on to receive cascade testing. Probability (b) was informed by the Panel Study of Income Dynamics (PSID), a longitudinal household survey[18]; from this we extracted the average number of surviving mothers, sisters, and

© 2020 Guzauskas GF et al. *JAMA Network Open.*

daughters by age per woman screened. Probabilities for (d) and (e) were equivalent to those used in the primary population screening model.

eFigure 1. Cascade Testing Module Decision Tree

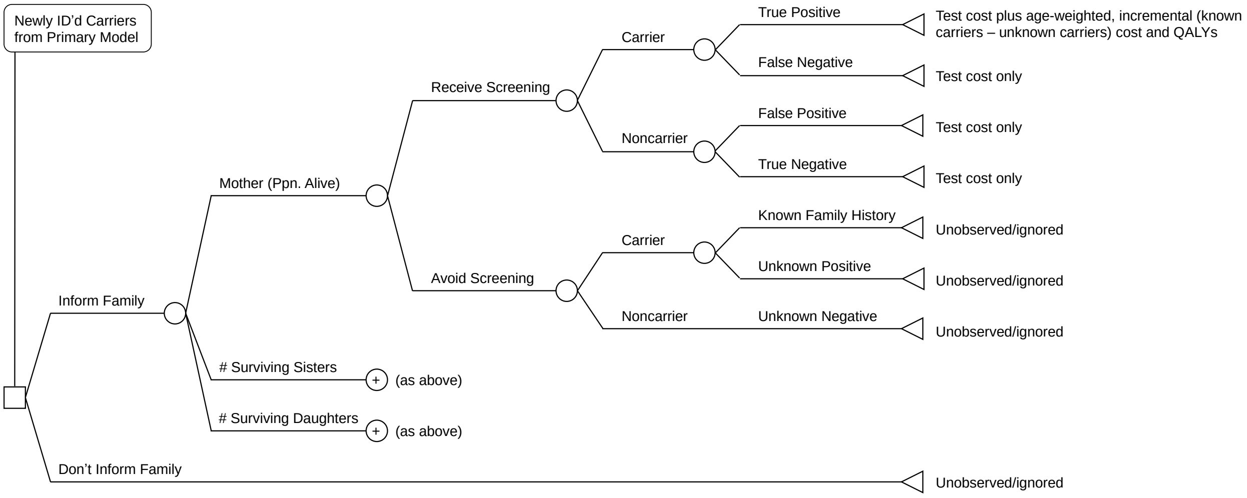

The cascade testing module utilized age-based results from the primary screening model, combined with publicly available data on the number of first-degree female relatives of each identified carrier,[18] to calculate the incremental cost and benefits and their impact on the overall model ICER. We estimated age-based incremental outcomes from ages 20 to 100, and estimated a weighted (by number and ages of mother, sisters, and daughters) sum of incremental cost and QALYs over all newly identified family members. These weighted incremental estimates were then "fed back" into the primary population screening model, such that the incremental outcomes from the cascade model are tied to the age of the patient entering the primary model. The process of using the primary model to inform cascade testing incremental outcomes, and then

© 2020 Guzauskas GF et al. *JAMA Network Open.*

adding back to the primary model for the cascade screening scenario, is depicted below.

eFigure 2. Calculation of Cascade Testing Outcomes

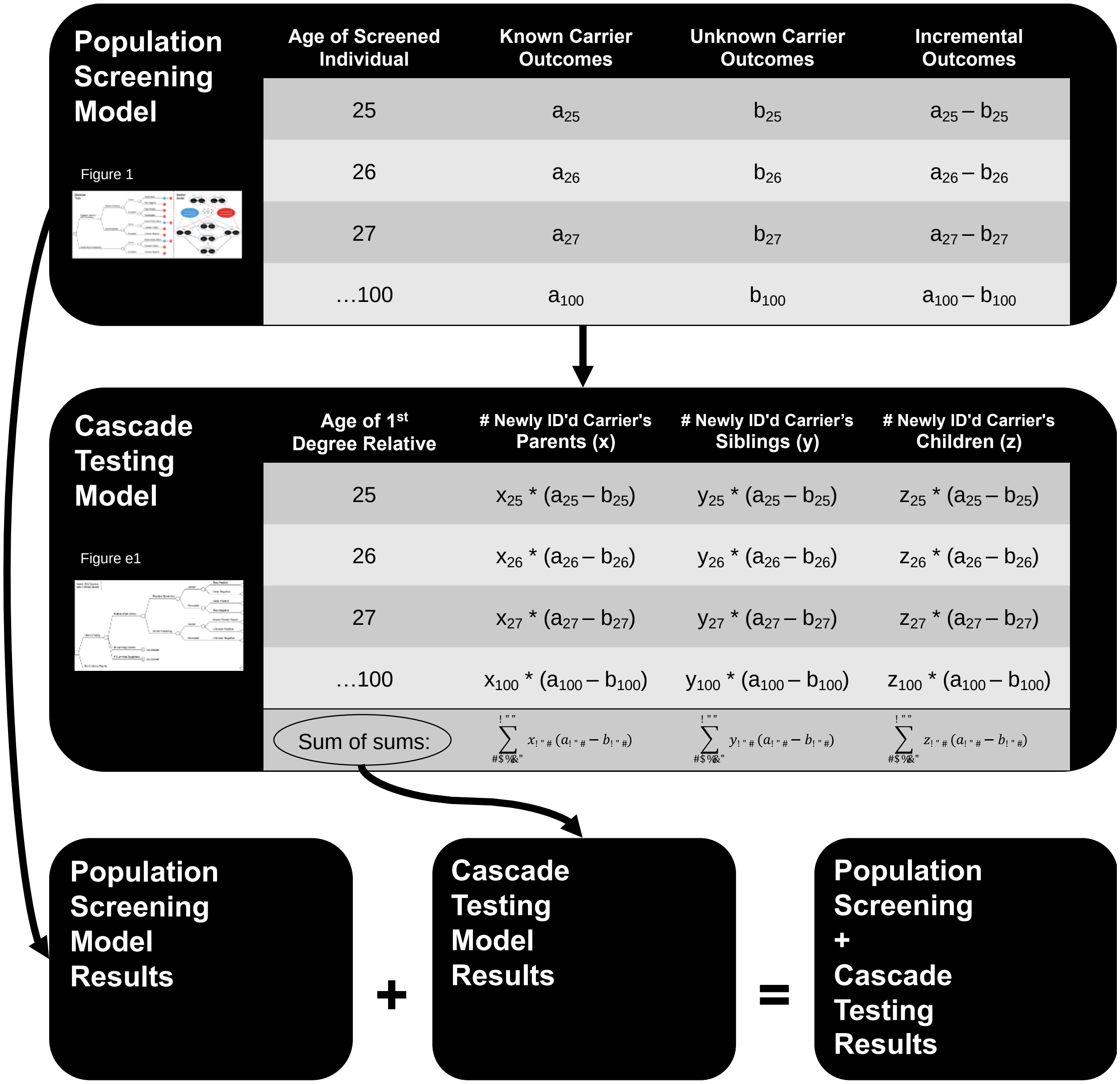

© 2020 Guzauskas GF et al. *JAMA Network Open.*

eTable. Expanded Model Results

	Incremental Results						
Screened Age	Earlier Stage Cases/100K	Later Stage Cases/100K	Total Cancer Cases/100K	Cost/ Woman Screened	QALYs/ Woman Screened	Life Years/ Woman Screened	ICER
20	125	-215	-90	$245	0.0027	0.0022	$90,600
21	126	-215	-89	$245	0.0028	0.0023	$89,100
22	127	-215	-88	$245	0.0028	0.0023	$87,600
23	128	-215	-87	$245	0.0028	0.0023	$86,200
24	128	-215	-86	$245	0.0029	0.0024	$84,900
25	131	-216	-86	$246	0.0029	0.0024	$83,800
26	132	-216	-83	$247	0.0029	0.0024	$84,200
27	134	-215	-81	$248	0.0029	0.0024	$84,700
28	135	-214	-79	$249	0.0029	0.0024	$85,400
29	137	-214	-77	$251	0.0029	0.0024	$86,500
**30**	**138**	**-214**	**-75**	**$252**	**0.0029**	**0.0024**	**$87,700**
31	140	-212	-72	$252	0.0028	0.0023	$90,700
32	143	-211	-68	$253	0.0027	0.0022	$94,400
33	145	-209	-64	$253	0.0026	0.0021	$99,200
34	147	-207	-60	$254	0.0024	0.0020	$105,300
35	150	-206	-56	$255	0.0023	0.0019	$112,500
36	152	-204	-52	$255	0.0022	0.0019	$118,100
37	154	-203	-49	$255	0.0020	0.0018	$125,200
38	155	-201	-46	$256	0.0019	0.0017	$134,300
39	157	-199	-42	$256	0.0017	0.0016	$146,900
40	157	-194	-37	$257	0.0016	0.0014	$164,100
41	157	-192	-35	$258	0.0015	0.0014	$175,800
42	158	-190	-32	$258	0.0014	0.0013	$190,400
43	158	-188	-30	$258	0.0012	0.0012	$209,200
44	159	-185	-27	$259	0.0011	0.0011	$234,500
**45**	**159**	**-183**	**-24**	**$261**	**0.0010**	**0.0010**	**$268,200**
46	159	-181	-22	$261	0.0009	0.0009	$291,300
47	159	-179	-20	$262	0.0008	0.0009	$320,800
48	159	-177	-18	$262	0.0007	0.0008	$359,800
49	159	-175	-16	$263	0.0006	0.0008	$415,100
50	159	-173	-14	$265	0.0005	0.0007	$482,100
51	157	-171	-13	$265	0.0005	0.0007	$512,600
52	156	-168	-13	$264	0.0005	0.0006	$549,200
53	154	-166	-12	$264	0.0004	0.0006	$593,900
54	153	-164	-11	$264	0.0004	0.0006	$649,700
55	148	-157	-9	$263	0.0003	0.0005	$831,500
56	146	-155	-8	$262	0.0003	0.0005	$889,700
57	144	-152	-8	$261	0.0003	0.0005	$961,100
58	143	-150	-7	$260	0.0002	0.0004	$1,051,100

© 2020 Guzauskas GF et al. *JAMA Network Open.*

Screened Age	Incremental Results Earlier Stage Cases/100K	Later Stage Cases/100K	Total Cancer Cases/100K	Cost/ Woman Screened	QALYs/ Woman Screened	Life Years/ Woman Screened	ICER
59	141	-148	-7	$259	0.0002	0.0004	$1,169,200
60	136	-141	-5	$257	0.0002	0.0004	$1,666,100
61	133	-138	-5	$256	0.0001	0.0004	$1,773,100
62	130	-136	-5	$255	0.0001	0.0003	$1,897,200
63	128	-133	-5	$254	0.0001	0.0003	$2,043,600
64	125	-130	-5	$253	0.0001	0.0003	$2,219,700
65	119	-123	-4	$250	0.0001	0.0003	$4,424,400
66	116	-120	-4	$249	0.0000	0.0003	$5,360,300
67	113	-116	-3	$248	0.0000	0.0003	$6,856,400
68	110	-113	-3	$246	0.0000	0.0002	$9,655,100
69	106	-110	-3	$245	0.0000	0.0002	$16,930,700
70	100	-102	-2	$243	-0.0001	0.0002	-$4,391,900
71	96	-98	-2	$242	-0.0001	0.0002	-$3,910,000
72	92	-94	-2	$241	-0.0001	0.0002	-$3,522,000
73	88	-90	-2	$239	-0.0001	0.0002	-$3,200,900
74	84	-85	-2	$238	-0.0001	0.0002	-$2,929,200
75	83	-84	-1	$239	-0.0001	0.0001	-$2,448,000
76	78	-79	-1	$237	-0.0001	0.0001	-$2,309,600
77	73	-75	-1	$236	-0.0001	0.0001	-$2,185,700
78	68	-70	-1	$235	-0.0001	0.0001	-$2,073,400
79	63	-64	-1	$234	-0.0001	0.0001	-$1,970,500
80	57	-58	-1	$231	-0.0002	0.0001	-$1,492,500
81	54	-55	-1	$230	-0.0002	0.0001	-$1,448,000
82	51	-52	-1	$228	-0.0002	0.0001	-$1,406,500
83	48	-49	-1	$227	-0.0002	0.0001	-$1,367,500
84	45	-46	-1	$225	-0.0002	0.0001	-$1,330,200
85	43	-44	-1	$224	-0.0002	0.0001	-$1,314,100
86	40	-41	-1	$223	-0.0002	0.0001	-$1,283,200
87	37	-37	-1	$221	-0.0002	0.0001	-$1,253,800
88	33	-34	-1	$220	-0.0002	0.0001	-$1,225,300
89	30	-30	-1	$219	-0.0002	0.0001	-$1,196,600
90	24	-25	0	$216	-0.0002	0.0000	-$1,037,300
91	23	-23	0	$214	-0.0002	0.0000	-$1,025,400
92	22	-22	0	$213	-0.0002	0.0000	-$1,014,100
93	21	-21	0	$212	-0.0002	0.0000	-$1,003,100
94	20	-20	0	$211	-0.0002	0.0000	-$991,800
95	15	-15	0	$208	-0.0002	0.0000	-$940,100
96	14	-14	0	$206	-0.0002	0.0000	-$930,000
97	12	-13	0	$205	-0.0002	0.0000	-$919,700
98	10	-10	0	$202	-0.0002	0.0000	-$908,500
99	7	-8	0	$199	-0.0002	0.0000	-$894,900

© 2020 Guzauskas GF et al. *JAMA Network Open.*

In one-way sensitivity analysis, one parameter at a time is varied to its low and high value while keeping all other parameters constant. Parameters with the greatest impact on results have the largest bars in the “tornado” diagram and are located on top.

eFigure 3. Results of One-Way Sensitivity Analysis

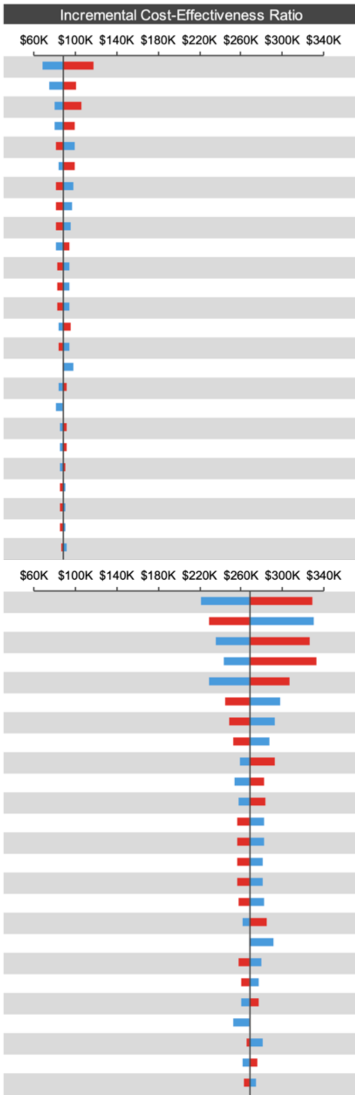

Parameter	Low Input	High Input	Low Result	High Result	Spread
**Population: 30-Year-Old Women**					
Breast Cancer 5-Year Survival	-20%	+20%	$68,291	$117,098	$48,806
Screening Assay Cost	$160	$240	$74,438	$100,866	$26,428
Breast Cancer HR Post-Oophorectomy: BRCA2	0.16	0.82	$79,879	$106,015	$26,136
Breast Cancer HR Post-Oophorectomy: BRCA1	0.41	0.96	$79,998	$99,216	$19,218
BRCA2 Oophorectomy Annual Uptake	-50%	+50%	$99,879	$80,808	$19,071
Ovarian Cancer HR Post-Oophorectomy: BRCA1	0.12	0.82	$83,332	$99,657	$16,325
BRCA1 Oophorectomy Annual Uptake	-50%	+50%	$97,705	$81,649	$16,056
Proportion of Carriers: BRCA2	0.232	0.348	$96,186	$80,563	$15,623
BRCA2 Mastectomy Annual Uptake	-50%	+50%	$95,302	$81,659	$13,643
Annual Intense Screening (MRI) Cost	$1,122	$1,683	$81,519	$93,785	$12,266
BRCA1 Mastectomy Annual Uptake	-50%	+50%	$94,345	$82,126	$12,219
HBOC Carrier Prevalence (Overall)	0.452%	0.538%	$94,014	$82,318	$11,696
Proportion of Carriers: BRCA1	0.221	0.332	$93,929	$82,250	$11,679
Breast Cancer HR Post-Oophorectomy: Non-BRCA	0.16	0.82	$84,192	$95,711	$11,518
BRCA2 Breast Cancer Annual Incidence	-20%	+20%	$93,476	$83,091	$10,385
Utility: Well	0.950	1.000	$97,388	$87,652	$9,737
Utility: Post Breast Cancer	0.770	0.851	$83,958	$91,686	$7,729
Mammography+MRI Uptake, Increased Risk, 30-74	0.800	1.000	$80,696	$87,652	$6,955
Proportion of Known Carriers Who Get MRI	0.600	0.900	$84,401	$90,867	$6,466
Breast Cancer Mortality Relative Reduction: Early Stage	0.900	1.000	$85,025	$91,227	$6,202
Proportion ID'd Mutations with FH Testing	0.139	0.209	$84,842	$90,709	$5,867
BRCA1 Breast Cancer Annual Incidence	-20%	+20%	$90,651	$85,263	$5,388
Breast Cancer Odds Ratio: PALB2	2.24	17.65	$90,249	$84,892	$5,357
General Population Breast Cancer Annual Incidence	-20%	+20%	$90,142	$85,553	$4,589
Screening Test Sensitivity	0.950	1.000	$91,405	$86,917	$4,488
**Population: 45-Year-Old Women**					
Breast Cancer 5-Year Survival	-20%	+20%	$221,245	$329,244	$107,999
BRCA2 Oophorectomy Annual Uptake	-50%	+50%	$329,820	$228,412	$101,408
Breast Cancer Mortality Relative Reduction: Early Stage	0.900	1.000	$235,115	$326,329	$91,214
Breast Cancer HR Post-Oophorectomy: BRCA2	0.16	0.82	$242,693	$333,326	$90,633
Screening Assay Cost	$160	$240	$229,033	$307,290	$78,257
Proportion of Carriers: BRCA2	0.232	0.348	$297,360	$244,649	$52,711
BRCA1 Oophorectomy Annual Uptake	-50%	+50%	$291,885	$248,740	$43,145
HBOC Carrier Prevalence (Overall)	0.452%	0.538%	$286,997	$252,369	$34,629
Ovarian Cancer HR Post-Oophorectomy: BRCA1	0.12	0.82	$259,153	$292,391	$33,238
Annual Intense Screening (MRI) Cost	$1,122	$1,683	$253,667	$282,656	$28,988
Breast Cancer HR Post-Oophorectomy: BRCA1	0.41	0.96	$257,221	$283,770	$26,549
Proportion of Carriers: BRCA1	0.221	0.332	$281,943	$255,961	$25,982
Cascade Screening: Proportion. Carriers	0.400	0.600	$281,510	$256,195	$25,315
Cascade Screening: Proportion. Tell Family	0.560	0.840	$281,441	$256,257	$25,184
Cascade Screening: Proportion. Family Tested	0.160	0.240	$281,441	$256,257	$25,184
BRCA2 Breast Cancer Annual Incidence	-20%	+20%	$282,360	$257,215	$25,145
Breast Cancer HR Post-Oophorectomy: Non-BRCA	0.16	0.82	$260,972	$284,644	$23,671
Utility: Well	0.950	1.000	$291,409	$268,162	$23,247
BRCA2 Mastectomy Annual Uptake	-50%	+50%	$279,792	$257,541	$22,251
BRCA1 Mastectomy Annual Uptake	-50%	+50%	$277,446	$259,541	$17,905
Proportion ID'd Mutations with FH Testing	0.139	0.209	$260,171	$276,830	$16,659
Mammography+MRI Uptake, Increased Risk, 30-74	0.800	1.000	$252,315	$268,162	$15,847
Screening Test Sensitivity	0.950	1.000	$281,387	$265,576	$15,811
Utility: Post Breast Cancer	0.770	0.851	$261,536	$275,132	$13,596
BRCA1 Breast Cancer Annual Incidence	-20%	+20%	$274,661	$262,817	$11,844

© 2020 Guzauskas GF et al. *JAMA Network Open.*

Probabilistic sensitivity analysis results are presented as cost-effectiveness acceptability curves, which show the Bayesian probability that the results are cost-effective at increasing willingness-to-pay per QALY thresholds (supplementary Figure e4). For 30-year-olds, population screening had a 0%, 78%, and 100% probability of being cost-effective versus family history testing alone at the $50,000, $100,000, and $150,000 per QALY thresholds, respectively. When we added potential harm to noncarriers who avoid recommended mammography, the probability of population screening being cost-effective fell to 0%, 2%, 34% at the $50,000, $100,000, and $150,000 per QALY thresholds, respectively. Population screening was not cost-effective for 45-year-olds in any probabilistic simulations with a willingness-to-pay per QALY threshold below $150,000.

eFigure 4. Results of Probabilistic Sensitivity Analysis

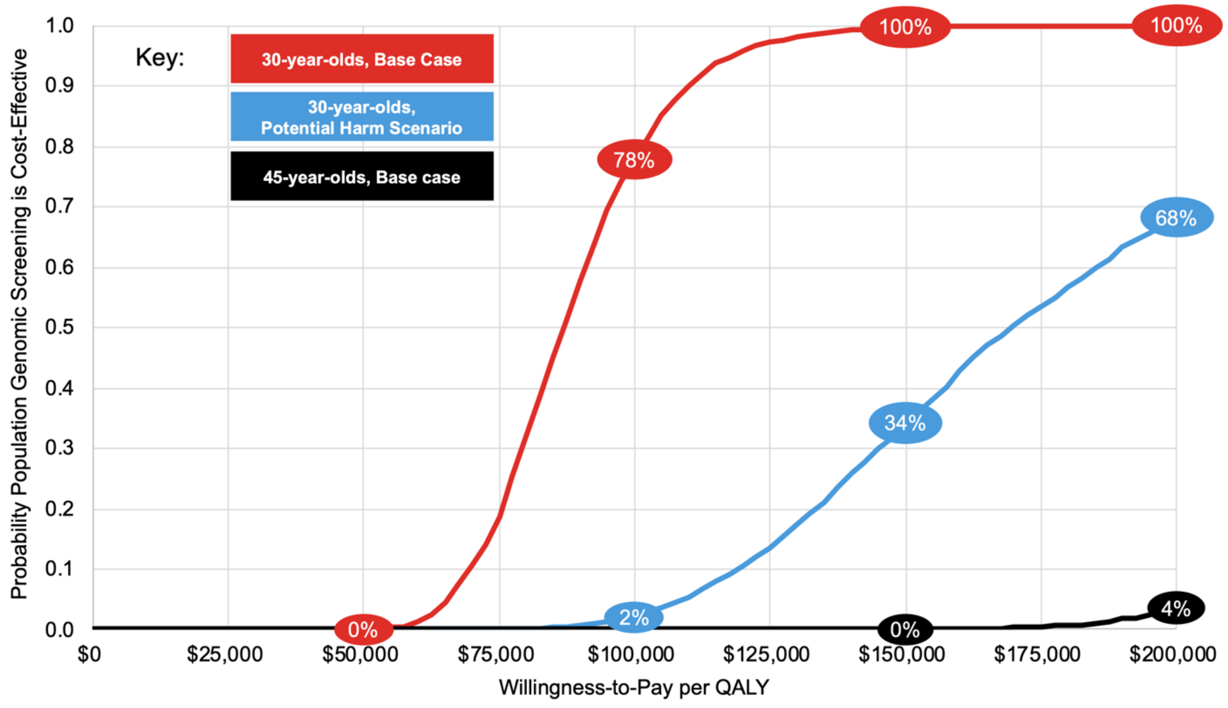

© 2020 Guzauskas GF et al. *JAMA Network Open.*

eReferences.

1. Dewey FE, Murray MF, Overton JD, et al. Distribution and clinical impact of functional variants in 50,726 whole-exome sequences from the DiscovEHR study. *Science*. Dec 23 2016;354(6319)doi:10.1126/science.aaf6814

2. Manickam K, Buchanan AH, Schwartz MLB, et al. Exome Sequencing-Based Screening for BRCA1/2 Expected Pathogenic Variants Among Adult Biobank Participants. *JAMA Netw Open*. Sep 7 2018;1(5):e182140. doi:10.1001/jamanetworkopen.2018.2140

3. Toland AE, Forman A, Couch FJ, et al. Clinical testing of BRCA1 and BRCA2: a worldwide snapshot of technological practices. *NPJ Genom Med*. 2018;3:7. doi:10.1038/s41525-018-0046-7

4. Manchanda R, Patel S, Gordeev VS, et al. Cost-effectiveness of Population-Based BRCA1, BRCA2, RAD51C, RAD51D, BRIP1, PALB2 Mutation Testing in Unselected General Population Women. *J Natl Cancer Inst*. Jul 1 2018;110(7):714-725. doi:10.1093/jnci/djx265

5. Kurian AW, Ward KC, Howlader N, et al. Genetic Testing and Results in a Population-Based Cohort of Breast Cancer Patients and Ovarian Cancer Patients. *J Clin Oncol*. May 20 2019;37(15):1305-1315. doi:10.1200/jco.18.01854

6. Daly MB, Pilarski R, Axilbund JE, et al. Genetic/Familial High-Risk Assessment: Breast and Ovarian, Version 2.2015. *J Natl Compr Canc Netw*. Feb 2016;14(2):153-62. doi:10.6004/jnccn.2016.0018

7. Robson ME, Bradbury AR, Arun B, et al. American Society of Clinical Oncology Policy Statement Update: Genetic and Genomic Testing for Cancer Susceptibility. *J Clin Oncol*. Nov 1 2015;33(31):3660-7. doi:10.1200/jco.2015.63.0996

8. Bevers TB, Helvie M, Bonaccio E, et al. Breast Cancer Screening and Diagnosis, Version 3.2018, NCCN Clinical Practice Guidelines in Oncology. *J Natl Compr Canc Netw*. Nov 2018;16(11):1362-1389. doi:10.6004/jnccn.2018.0083

9. Morgan RJ, Jr., Armstrong DK, Alvarez RD, et al. Ovarian Cancer, Version 1.2016, NCCN Clinical Practice Guidelines in Oncology. *J Natl Compr Canc Netw*. Sep 2016;14(9):1134-63. doi:10.6004/jnccn.2016.0122

10. Chai X, Friebel TM, Singer CF, et al. Use of risk-reducing surgeries in a prospective cohort of 1,499 BRCA1 and BRCA2 mutation carriers. *Breast Cancer Res Treat*. Nov 2014;148(2):397-406. doi:10.1007/s10549-014-3134-0

© 2020 Guzauskas GF et al. *JAMA Network Open.*

11. Domchek SM, Friebel TM, Singer CF, et al. Association of risk-reducing surgery in BRCA1 or BRCA2 mutation carriers with cancer risk and mortality. *Jama*. Sep 1 2010;304(9):967-75. doi:10.1001/jama.2010.1237

12. Kuchenbaecker KB, Hopper JL, Barnes DR, et al. Risks of Breast, Ovarian, and Contralateral Breast Cancer for BRCA1 and BRCA2 Mutation Carriers. *Jama*. Jun 20 2017;317(23):2402-2416. doi:10.1001/jama.2017.7112

13. Lu HM, Li S, Black MH, et al. Association of Breast and Ovarian Cancers With Predisposition Genes Identified by Large-Scale Sequencing. *JAMA Oncol*. Jan 1 2019;5(1):51-57. doi:10.1001/jamaoncol.2018.2956

14. Surveillance Epidemiology and End Results (SEER) Program. Accessed 9/19/2019, https://seer.cancer.gov/statfacts/html/ovary.html

15. Evans DG, Kesavan N, Lim Y, et al. MRI breast screening in high-risk women: cancer detection and survival analysis. *Breast Cancer Res Treat*. Jun 2014;145(3):663-72. doi:10.1007/s10549-014-2931-9

16. Elrick A, Ashida S, Ivanovich J, et al. Psychosocial and Clinical Factors Associated with Family Communication of Cancer Genetic Test Results among Women Diagnosed with Breast Cancer at a Young Age. *J Genet Couns*. Feb 2017;26(1):173-181. doi:10.1007/s10897-016-9995-0

17. Roberts MC, Dotson WD, DeVore CS, et al. Delivery Of Cascade Screening For Hereditary Conditions: A Scoping Review Of The Literature. *Health Aff (Millwood)*. May 2018;37(5):801-808. doi:10.1377/hlthaff.2017.1630

18. Panel Study of Income Dynamics: Public use dataset. Produced and distributed by the Survey Research Center, Institute for Social Research, University of Michigan, Ann Arbor, MI. https://simba.isr.umich.edu/data/data.aspx

© 2020 Guzauskas GF et al. *JAMA Network Open.*
